# MmWave Physical Layer Network Modeling and Planning for Fixed Wireless Access Applications

**DOI:** 10.3390/s23042280

**Published:** 2023-02-17

**Authors:** Brecht De Beelde, Mike Vantorre, German Castellanos, Mario Pickavet, Wout Joseph

**Affiliations:** Department of Information Technology, Ghent University/IMEC, 9052 Gent, Belgium

**Keywords:** channel modeling, fixed wireless access FWA, mmWave, network design, routing algorithm, graphs

## Abstract

The large bandwidths that are available at millimeter-wave frequencies enable fixed wireless access (FWA) applications, in which fixed point-to-point wireless links are used to provide internet connectivity. In FWA networks, a wireless mesh is created and data are routed from the customer premises equipment (CPE) towards the point of presence (POP), which is the interface with the wired internet infrastructure. The performance of the wireless links depends on the radio propagation characteristics, as well as the wireless technology that is used. The radio propagation characteristics depend on the environment and on the considered frequency. In this work, we analyzed the network characteristics of FWA networks using radio propagation models for different wireless technologies using millimeter-wave (mmWave) frequencies of 28 GHz, 60 GHz, and 140 GHz. Different scenarios and environments were considered, and the influence of rain, vegetation, and the number of subscribers was investigated. A network planning algorithm is presented that defines a route for each CPE towards the POP based on a predefined location of customer devices and considering the available capacity of the wireless links. Rain does not have a considerable effect on the system capacity. Even though the higher frequencies exhibit a larger path loss, resulting in a lower power of the received signal, the larger bandwidths enable a higher channel capacity.

## 1. Introduction

During the past decade, the need for broadband connectivity has increased. Not only do end-users require more data volumes and higher data rates, e.g., for online gaming and video-on-demand streaming services, but also the data volumes of enterprises have risen, e.g., due to digitalization, video conferencing, and telework [1]. As the capacity of the current wired infrastructure is limited, e.g., the data rate that can be obtained using digital subscriber line (DSL) technology is generally limited to 100 Mbps [2], network operators are required to update their access networks to enable broadband networks. Fiber optic cables offer download speeds of up to 10 Gbps [3] but have a high installation cost [4]. In fixed wireless access (FWA) applications, the last mile of the access network is replaced by a wireless link using a point-to-point radio network. The large bandwidths that are available at millimeter-wave (mmWave) frequencies enable broadband access networks [5] with a lower installation cost compared to the deployment of a fiber network, as no costly digging is required [4]. The narrow beamwidth antennas, enabled by large antenna arrays, allow for spatial filtering, which limits interference in dense networks.

In this paper, we characterized FWA networks via a graph analysis using state-of-the-art radio propagation models, and we present a tool used to perform the network planning of FWA networks. Different scenarios and environments were considered, and network capacity calculations were performed for different wireless technologies envisioned for FWA networks, i.e., fifth-generation (5G) communication in the 28 GHz band, and IEEE Std. 802.11ad in the 60 GHz band. We also performed capacity calculations for the 140 GHz band that is envisioned for future wireless communication systems.

The outline of this paper is as follows. In Section 2, we describe the envisioned FWA network architecture and we present an overview of the different radio propagation models that are used in this work. This is followed in Section 3 by the methodology of how the network analysis and planning are performed. We also present how network data are obtained via simulations to model different types of FWA networks. In Section 4.1, the FWA network analysis is presented, and Section 4.2 presents the network planning results. Section 5 concludes this paper. The implementation details of the network modeling and planning tool are discussed in the Appendix A.

### 1.1. Related Research

A tutorial on technologies and design considerations for FWA networks is provided in [6]. In [7], a mathematical model is presented for the automatic selection and configuration of base stations for FWA networks with wireless technologies using a carrier frequency of 3.5 GHz. An FWA network design using 5G technology is discussed in [8], and beam alignment at mmWave frequencies is discussed in [9]. In [10], capacity and coverage calculations for FWA using 5G technology in the 3.5 GHz and 28 GHz bands are presented. A study on the probability of signal outage probabilities for wireless backhaul communication at 28 GHz and 73 GHz is presented in [11]. In [5], a propagation model is provided for suburban FWA networks at 28 GHz with 90% coverage. Outdoor channel models for FWA applications are presented in [12] for wireless technologies at 60 GHz and in [13] for 140 GHz. The use of IEEE Std. 802.11ay for FWA networks is studied in [14]. Link budget calculations for FWA networks are presented in [15] for links using carrier frequencies ranging from 75 GHz to 400 GHz, and in [16] for frequencies from 300 GHz to 900 GHz. A study on FWA network deployments using IEEE Std. 802.11ay is presented in [17].

### 1.2. Contributions

In this work, we present a study on FWA network characterization and planning for different environments and scenarios. We considered frequency bands that are used in existing wireless technologies, i.e., the 28 GHz band used in mmWave 5G and the 60 GHz industrial, scientific, and medical (ISM) band used in IEEE Std. 802.11ad, as well as a frequency band at 140 GHz that could be used for future wireless communication systems. We used state-of-the-art radio propagation models to calculate the capacity of the wireless links for different scenarios. To the best of the authors’ knowledge, it is the first time that system-level performance simulations have been performed on a wireless communication system using a carrier frequency of 140 GHz. We also present a framework used to analyze FWA network characteristics and to perform network planning. The framework was implemented in Python and uses graph theory to define the location of routing devices. It implements an algorithm that routes traffic from all customer premises equipment (cpe) devices towards a connection to the wired backbone, considering the available data rates on the wireless links.

This work is an extension of our previous work [17]. Compared to [17], a different FWA network architecture is envisioned, and different network parameters are considered, i.e., in this paper, we compare different technologies and frequency bands.

## 2. Background Theory and Modeling

In this section, background theory and system-level aspects of FWA networks are provided, and radio propagation models and link budget calculations are presented.

### 2.1. Fixed Wireless Access Background

#### 2.1.1. Architecture Overview

**Figure 1:**
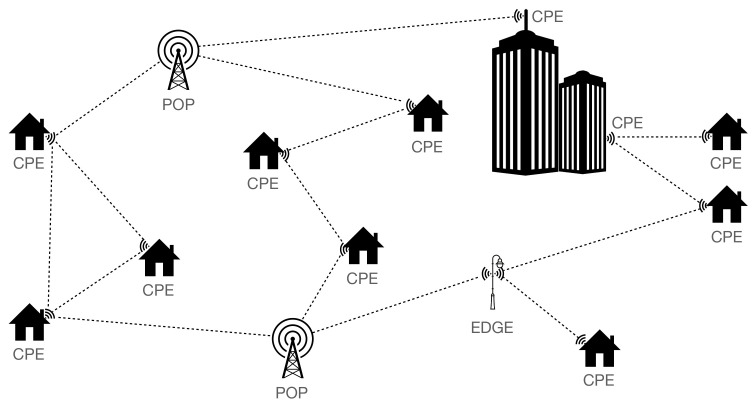
Fixed wireless access (FWA) system overview, with customer premises equipment (cpe) devices mounted on building facades forming a wireless mesh network. The mesh network is enabled by EDGE devices and the point of presence (pop) devices that form the interface to the wired internet infrastructure.

In FWA networks, internet connectivity is provided to residential and enterprise buildings via fixed wireless links, i.e., static wireless links are set up between multiple wireless devices [6]. An overview of the architecture of an FWA network is presented in Figure 1. Different types of devices are present in the network. Customer premises equipment (cpe) devices bridge the FWA network to the local area network (LAN) of the industrial or residential customer.

The cpe devices are typically connected at building facades above street level to limit attenuation due to moving people and cars. The number and location of cpe devices depend on the customers that subscribe to the FWA network and cannot be controlled by the network operator. The point of presence (pop) device forms the interface of the FWA network with the wired (backbone) infrastructure. There can be multiple pop devices, which increases the FWA network robustness, and, in most cases, the location of the pop devices is also predefined, e.g., based on the available wired backbone infrastructure. In order to connect to the network, all cpe devices need a connection to a pop device. By placing additional edge devices, a wireless mesh network is created that enables a connection from each cpe in the network towards a pop. The edge devices do not directly connect a customer to the network, but they have networking capabilities to route data towards a pop device. They can be installed on public buildings or street furniture, including lamp posts and street signs.

Different FWA architectures exist. In [17], cpe devices do not have networking capabilities and they can only connect to edge devices. The benefit of this architecture is that the cpe devices are cheaper to deploy, but the number of required edge devices increases. In this work, we considered an architecture where cpe devices have networking capabilities and can route data from a neighboring cpe or edge device to another device. It is still possible that, given predefined cpe and pop locations, some cpe devices are not connected to a pop because they have no neighboring devices to which they can connect or because the capacity of the wireless links is not sufficient for transferring the data of all cpe devices that use the link. To create a mesh network where all cpe devices get connected to a pop device, edge devices might be added to the FWA network. They enable the connectivity of cpe devices and increase the network capacity. As such, the (only) differences between edge and cpe devices are the following:

1. cpe devices connect customers, whereas edge devices only forward data.
2. The location of cpe devices is determined by the customers that subscribe to the network, whereas the location of edge devices is determined by the network operator.

#### 2.1.2. Standardization

Work on the standardization of broadband wireless access systems started two decades ago and resulted in IEEE Std. 802.16, which specifies the air interface, including the medium access control layer (MAC) and physical layer (PHY) of fixed and mobile point-to-multipoint broadband wireless access systems [18]. The IEEE Std. 802.16 considers frequencies ranging from 10 GHz to 66 GHz using channel bandwidths of 25 MHz and maximum data rates of 120 Mbps. This is five times the data rate of 24.4 Mbps for a single-input single-output (SISO) LTE system with quadrature amplitude modulation (256-QAM) and 5 MHz channel bandwidths, which follows the Shannon capacity theorem. Recent advancements in radio technology have realized mmWave radio communication for frequencies up to 100 GHz [19,20], and the higher available bandwidths enable high-throughput wireless communication systems. The IEEE Std. 802.11ad specifies PHY and MAC layer interfaces for short-range high-throughput wireless systems with carrier frequencies in the V-band (50–75 GHz) and channel bandwidths of 2 GHz, allowing for SISO data rates up to 4.6 Gbps [21]. Its successor, IEEE Std. 802.11ay, supports multiple-input multiple-output (MIMO) systems and allows for data rates up to 40 Gbps [22].

#### 2.1.3. Antenna and Transceiver Considerations

The small wavelengths at mmWave frequencies allow for systems with large antenna arrays, which result in highly directive antennas and facilitating beamforming. In the next section, path loss (PL) at mmWave frequencies is discussed, and it will be clear that high-gain antennas are required to overcome the high PL. Furthermore, beamforming results in spatial filtering, which limits interference in dense networks. A review on mmWave antennas for systems in the 60 GHz frequency band is presented in [23]. A distributed antenna system for mmWave communication is presented in [24].

New technology nodes, e.g., the 90/65 nm complementary metal–oxide–semiconductor (CMOS) process, enable a high cutoff and oscillation frequencies that realize mmWave integrated circuits and transceivers [25]. Using 12 nm fin field-effect transistor (FinFET) technology, a maximum oscillation frequency of 315 GHz is reported [26]. In [27], a survey of mmWave transceivers is presented, and the architectural and circuit considerations for 5G mmWave transceivers are discussed. In [28], a system design is presented for IEEE Std. 802.11ad networks. In [25], a 60 GHz transceiver in 90 nm CMOS is presented for IEEE Std. 802.11ad applications based on a sliding intermediate frequency (IF) architecture. A V-band transceiver module with an integrated phased antenna array is presented in [29], reporting an antenna gain of up to 26 dBi.

Multiple papers on the design of power amplifiers and voltage controlled oscillators (VCOs) are published in the Special Issue on *5G front-end transceivers* [30]. The design of a 48 GHz phase-locked loop for 60 GHz transceivers [31] is presented in the Special Issue on *mmWave integrated circuits and systems for 5G applications* [32]. This Special Issue also covers other design aspects of mmWave transceivers, including bandpass filter design [33], variable gain amplifiers [34,35], and mixers [36].

### 2.2. Radio Propagation Models

Radio propagation models characterize how electromagnetic waves propagate in a certain environment. They depend on not only the specific environment but also the carrier frequency. In the following sections, different propagation mechanisms are discussed that influence FWA networks, and mmWave channel models are presented.

#### 2.2.1. Path Loss

Path loss (PL) is the signal attenuation between a transmitting and receiving antenna due to the spherical expansion of a waveform. In a free space environment, the electromagnetic wave does not interact with any objects, and the PL depends on the distance, as well as the carrier frequency of the wireless technology. PL (in dB) in a free space environment is calculated via (1), with *f* the frequency (in Hz), *d* the distance (in meter), and c the speed of light in air, i.e., c = 3 $\cdot 10^{8}$ m/s.

(1) $$\mathrm{PL}\left(f,d\right)=20\;\log_{10}\left(4\pi d\frac{f}{c}\right)$$

PL increases with distance and frequency. For a link with a distance of 100 m, PL in free space is 101.4 dB at 28 GHz, 108.0 dB at 60 GHz, and 115.4 dB at 140 GHz. In realistic environments, object interactions may occur, and PL is often modeled using empirical channel models, e.g., using the one-slope model from (2), with PL${}_{0}$ the PL in dB at reference distance d${}_{0}$ equal to 1 m, and n the dimensionless PL exponent that defines the distance dependence. The shadow margin $\chi_{\sigma }$ is based on a normal distribution with standard deviation σ in dB [37].

(2) $$\mathrm{PL}\left(f,d\right)={\mathrm{PL}}_{0}\left(f,d_{0}\right)+10n\log_{10}\left(\frac{d}{d_{0}}\right)+\chi_{\sigma }$$

The model parameters PL${}_{0}$, n, and σ are fitted based on measurement data. In line-of-sight (LOS) scenarios, no objects are present in the first Fresnel zone of the link between the two antennas, and the direct path is unobstructed. The fitted parameters for LOS scenarios at different frequencies are presented in Table 1. At mmWave frequencies, limited interactions occur, and the fitted parameters are close to the free space scenario. Due to the highly directive antennas used in FWA systems, free space PL is a good representation in the case of an unobstructed line-of-sight path [12,13,38].

#### 2.2.2. Atmospheric Absorption

Radio waves at mmWave frequencies do not only attenuate due to the spreading of the wavefront: attenuation is also caused by absorption by atmospheric gases, i.e., oxygen and water vapor molecules [39]. As the gaseous component has a set of spectral absorption lines, the atmospheric attenuation is frequency-dependent [40].

In this work, the International Telecommunication Union (ITU) recommendation on attenuation by atmospheric gases and related effects was used to obtain attenuation values for the different frequency bands [41]. The specific attenuation in dB/km was predicted for an air pressure of 1013.25 hPa, a temperature of 15 °C, and a water vapor density of 7.5 g/m${}^{3}$. There is a peak in attenuation at sea level at 60 GHz, with a specific attenuation of 20 dB/km [42]. At 28 GHz (0.06 dB/km) and 140 GHz (0.4 dB/km), the attenuation is limited.

The one-slope PL model parameters from Table 1 include the atmospheric absorption, but the effect of the atmospheric absorption is limited due to the relatively small antenna separations.

#### 2.2.3. Rain Attenuation

The wavelengths corresponding to mmWave frequencies range from 10.7 mm at 28 GHz to 1 mm at 300 GHz, while the diameter of raindrops is of the order of 1 to 10 mm. Therefore, electromagnetic waves incident on raindrops will suffer from attenuation and scattering, in addition to the permittivity of water, which also differs from free space [43]. As such, the received signal strength will decrease in the event of rain, causing a lower link capacity.

Multiple models are available for predicting attenuation due to rain [43,44,45]. We used the recommendation from the ITU, which predicts the specific attenuation γ in dB/km via (3), with *R* the rain rate in mm/h and k and α frequency-dependent coefficients that are derived via a scattering analysis [46].

(3) $$\gamma \left(R\right)=kR^{\alpha }$$

Table 2 presents the specific attenuation for two rain rate intensities at different frequencies. The specific attenuation for a rain rate of 25 mm/h ranges from 3.9 dB/km at 28 GHz up to 6.8 dB/km at 60 GHz and 12.6 dB/km at 140 GHz.

#### 2.2.4. Vegetation Loss

In FWA networks, antennas are mounted at the building facades and above street level to limit link obstructions by vehicles and other objects. However, it is possible that trees obstruct the LOS path between two devices. Therefore, we need to take into account the attenuation due to vegetation obstructing the wireless link.

Multiple models are available to estimate vegetation loss as a function of frequency and vegetation depth [47,48,49,50,51]. These models have the generic form of (4), with *f* the frequency in MHz or GHz and *d* the vegetation depth in meters. Model parameters A, B, and C are estimated from measurement data.

(4) $$L\left(f,d\right)\left[\mathrm{dB}\right]=Af^{B}d^{C}$$

The Weissberger [47] and ITU-R [48] models are applicable for frequencies of up to 95 GHz. The COST-235 model [49] is applicable for frequencies of up to 57 GHz, and the FITU-R model [52] is applicable for frequencies of up to 40 GHz. In [53], the COST-235 model provides the best fit to measured vegetation loss at 60 GHz. Parameter B of the COST-235 model has a negative value of −0.009, and the vegetation loss decreases with an increasing frequency, as the smaller Fresnel radius at higher frequencies allows for radio propagation via the gaps in the vegetation [50]. The vegetation-dependent exponential decay (VED) model from [50] takes the vegetation density into account and is applicable for frequencies in the D-band, which includes 140 GHz.

For the remainder of this paper, the COST-235 model is used at frequencies of 28 GHz and 60 GHz, and the VED model is used at the frequency of 140 GHz. The estimated vegetation loss for a vegetation depth of 10 m is 25.9 dB at 28 GHz, 25.7 dB at 60 GHz, and 15.2 dB at 140 GHz.

### 2.3. Link Budget Calculation

A link budget calculation allows for determining the maximum data rate and range of a wireless system. It is based on configuration parameters, including the used wireless technology and antenna characteristics, as well as the propagation model. We calculated the received power (P${}_{R}$) in dBm of the electromagnetic wave using the link budget equation presented in (5), in which P${}_{T}$ is the transmit power in dBm, G${}_{T}$ and G${}_{R}$ are the antenna gains in dBi of the transmitting and receiving antenna systems (including the array gain), L${}_{T}$ is the feeder loss in dB at the transmitting device, L${}_{R}$ is the loss in dB at the receiving device, and PL is the path loss in dB, which includes environmental and atmospheric losses.

(5) $$P_{R}=P_{T}+G_{T}+G_{R}-L_{T}-L_{R}-\mathrm{PL}$$

With higher received powers, more complex modulation and coding schemes (MCSs) can be used, resulting in higher throughputs. The receiver sensitivity P${}_{\mathrm{RS}}$ (in dBm) is the minimum received power that is required in order to use a certain MCS, and it depends on the wireless technology and on the used MCS. The received power should be higher than the receiver sensitivity to use a certain MCS and to achieve the corresponding data rate.

The IEEE Std. 802.11ad specification [21] lists the minimum receiver sensitivities for the different MCSs, ranging from P${}_{\mathrm{RS}}$ = −68 dBm for MCS 1, which enables a maximum data rate of 385 Mbps, up to P${}_{\mathrm{RS}}$ = −53 dBm for MCS 12, which has a data rate of 4.62 Gbps, using a single carrier physical layer.

## 3. Methodology

In this section, we describe the methodology for the FWA network characterization and present the proposed network planning algorithm. Furthermore, we present how FWA network data are obtained via simulations, and we discuss the scenarios that are considered for the network characterization and the validation of the network planning algorithm.

### 3.1. Network Analysis

We use graph theory to analyze FWA networks. A graph *g* consists of a collection of vertices *v* that represent the devices in the network and a collection of edges *e* that connect two vertices [54]. The FWA network topology is represented by a graph where devices are represented by vertices and where edges indicate that a line-of-sight path exists between two devices, i.e., no buildings are obstructing the direct path between the two devices. The vertices have attributes, e.g., indicating the device type and location of the device that it represents. The edges have the link distance as an attribute. We analyzed the following metrics for the different networks.

**Average cpe vertex degree** $\delta_{v,\mathrm{avg}}$ **:**
 The average number of links of all cpe devices;
**POP eccentricity** $\epsilon_{\mathrm{POP}}$ **:**
 Maximum of the shortest distance from the pop to all other cpe devices in the graph;
**Median link length d** ${}_{\mathrm{med}}$ **:**
 Median distance in meters between two devices;
**Average path length l** ${}_{\mathrm{avg}}$ **:**
 Average hop count of the shortest path length of all cpe devices towards a pop device;
**Total network capacity:**  The total capacity of the network that is available on all wireless links.

These metrics influence the performance and quality of service (QoS) of the network. With a higher average cpe vertex degree, the network density increases. With more possible links between devices, the total network capacity increases, and fewer edge devices are required to obtain a route from each cpe device towards a pop device. The average link length, measured in meters, also gives an indication of the network capacity, as wireless links with a smaller link distance have an increased signal-to-noise ratio (SNR) and more complex MCSs can be used, which results in a higher capacity. The average path length, measured in hop count, influences the network performance on a higher level. As radio propagation in free space travels at the speed of light, propagation delays are minimal, and latency and jitter are mainly caused by the MAC and network layer settings. With a higher number of hops on the path, the packet latency will also increase due to an increased processing time at the hops. Therefore, the latency in dense networks is expected to be smaller than for the field trial and early adopter scenarios.

The analysis was conducted using Python’s igraph package, and the validation of our analysis scripts was performed using a simplified small FWA network for which we can easily calculate graph statistics manually. The results of the network analysis are presented in Section 4.1.

### 3.2. Network Planning

The goal of the network planning algorithm is twofold. First, the locations of edge devices need to be defined in order to get all cpe devices connected to the FWA network. Second, each cpe device needs to have a route with a sufficient capacity towards a pop device. Edge devices do not connect any customers directly and are added to the network for two reasons. First, they can be used to create a wireless mesh, i.e., connect cpe devices that can otherwise not connect to the FWA network. Second, they increase the network capacity, e.g., when the capacity of a wireless link is not sufficient for transferring the required data.

During the network planning phase, a route is defined for each cpe towards a pop, given all cpe and pop device locations, and a predefined QoS requirement, i.e., we need to allocate a certain data rate for each cpe on the wireless links that are used to reach the pop device.

#### 3.2.1. Prerequisites

The required input data for the network planning algorithm is a database containing the LOS links between all devices, as well as the link distances in meters. The link budget parameters from Section 2.3 are configured, including a constant antenna gain (independent of the beamforming angle), the rain rate, and possible vegetation obstructing the LOS path. No reflected paths are considered. Furthermore, each cpe device has an associated data rate requirement. In Section 3.3, the methodology for obtaining the database with wireless link info is described.

#### 3.2.2. Preparation

From the database, a graph was constructed where vertices represent devices, with device type (cpe, pop, or edge), data rate requirement (for cpe devices), and physical location as attributes. In the graph, edges represent that a LOS path is present between two devices, with the distance (in meters) as an attribute. From the distance attributes, wireless link capacities were calculated based on the link budget calculation presented in Section 2.3 and added as an additional attribute. One additional (artificial) pop device was added, which was connected to all other pop devices. This allows for network topologies with multiple pop devices. The capacity of a link connecting a pop device to the artificial parent pop is the sum of all capacities of the child pop device.

Feasibility checks were performed on the input data before running the network planning algorithm. A first check consists of verifying that the input graph is connected, i.e., there is a path from any vertex to any other vertex in the graph. If not, it is impossible to serve the unconnected cpe devices as no path towards a pop exists. A second feasibility check consists of summing the required data rates of all cpe devices and verifying that this is smaller than the sum of capacities of the links towards the artificial parent pop. If this is not the case, there is a capacity constraint at the pop devices and not all cpe devices will have their required data rate at peak moments. If one of these feasibility checks fails, a manual interaction is required, during which edge devices are added to the network, as we will describe in the next section. This manual interaction in network planning is also required to apply with (local) regulations [55]. If the capacity of the wireless links of the pop is not sufficient, additional edge devices can be added near the pop device, or additional pop devices can be added. This is again a manual decision based on the available infrastructure.

#### 3.2.3. Algorithm

Algorithm 1 describes the network planning algorithm based on an input graph with vertices that represent cpe, edge, and pop devices and edges that represent LOS links between devices. In the first step, the cpe devices are sorted. For all vertices with a cpe type attribute, the shortest paths towards the artificial pop are calculated via Dijkstra’s algorithm [54] using link distances as weights. The vertices are sorted in the following order:

1. The required data rate;
2. The number of shortest paths;
3. The number of hops on the shortest path.

**Algorithm 1:** Network routing for predefined throughput requirements. 
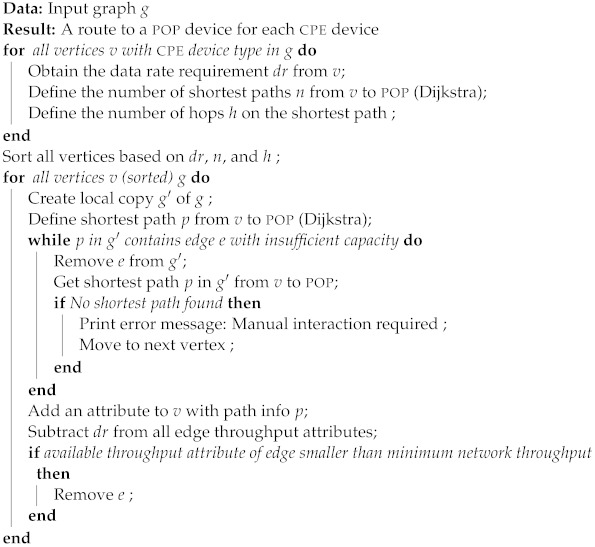


We first performed routing for the vertices with the highest data rate requirement in order to prevent first optimizing routes of other vertices and having no more link capacity available to serve the high-demanding customers. For vertices with identical data rate requirements, routing was first performed for vertices with the lowest number of shortest paths towards the (parent) pop device. Lastly, for vertices with equal data rate requirements and the number of shortest paths, we performed routing first for the vertices with the highest number of hops on the shortest path.

After having a sorted list of vertices for which we need to define routing towards the pop device, we again used the shortest path algorithm to define the routing. An attribute was added to the vertex with the path that needs to be followed, and the available link capacity attribute was updated on all edges along that path, i.e., the “available” remaining data rate of the wireless link was lowered by the throughput requirement of the vertex. If the available data rate on an edge was lower than the data rate requirement of the network, we ran the shortest path algorithm again after temporarily removing the edge from the graph. If the available data rate on an edge was lower than the minimum data rate requirement of the network, we removed the edge to prevent this edge from being used for routing traffic of other vertices. Therefore, the graph gets updated and the shortest path algorithm will result in other paths compared to the first time we ran the algorithm.

If the preparation or network planning failed, e.g., due to the graph non-connectivity or due to a throughput bottleneck on one of the wireless links, a manual intervention was required, in which additional edge devices were placed in the network. The determination of the location of the edge devices is a manual task that is difficult to automate due to the high number of legal and practical restrictions where edge devices might be placed, i.e., the placement of base stations is subject to regulations [56]. Furthermore, the number of edge devices in the network is expected to be limited. The locations of the additional edge devices were added to the input database, as well as the LOS links, and their corresponding distances to other devices in the network.

The algorithm was implemented in Python. A presentation of the network characterization and network planning tool is provided in the Appendix A.

### 3.3. Network Data Acquisition

We obtained FWA network data from simulations using the green radio access network design (GRAND) tool, which is a deployment tool for wireless radio access networks [57]. The tool is designed for the network planning of cellular networks, i.e., to define base station locations considering mobile users, and has been adjusted to enable the simulation of FWA networks [58].

The starting point of the tool is a map of the considered deployment environment. The map consists of three parts: the building locations, the street locations, and the area limits. All of the buildings obtain a unique building identifier. In Belgium, this map is freely available from the government or from OpenStreetMap [59]. A configurable number of cpe devices were added to this map, with the constraints that there can be at most one device per building and that the device is positioned at the building’s facade near the street. The Cartesian coordinates of these devices were randomly defined via a uniform distribution using the area limits from the map. For each device, new coordinates were generated as long as the coordinates did not correspond with a building that has no cpe device already assigned. Once the coordinates corresponded to a building, they were modified so that the cpe was located at the street-level facade of the building at a height of 4 m above the ground. The result of the cpe device placement algorithm is that a predefined number of devices are randomly allocated to different buildings on the map, at the facade closest to the nearest street. From the GRAND tool, we obtained a database with identifiers and coordinates of all cpe devices. In the second step, the possible radio links between different cpe devices were defined by searching for possible LOS paths between two devices based on the location of devices and the map with building locations. From this second step, we obtained a database with all LOS links between the different cpe devices. For each link, we obtained the identifiers of the two devices, as well as the link distance in meters.

By selecting different input maps, network data for different environments were generated. By adjusting the number of cpe devices that are present in the network, different scenarios were simulated.

### 3.4. Considered Scenarios

**Figure 2:**
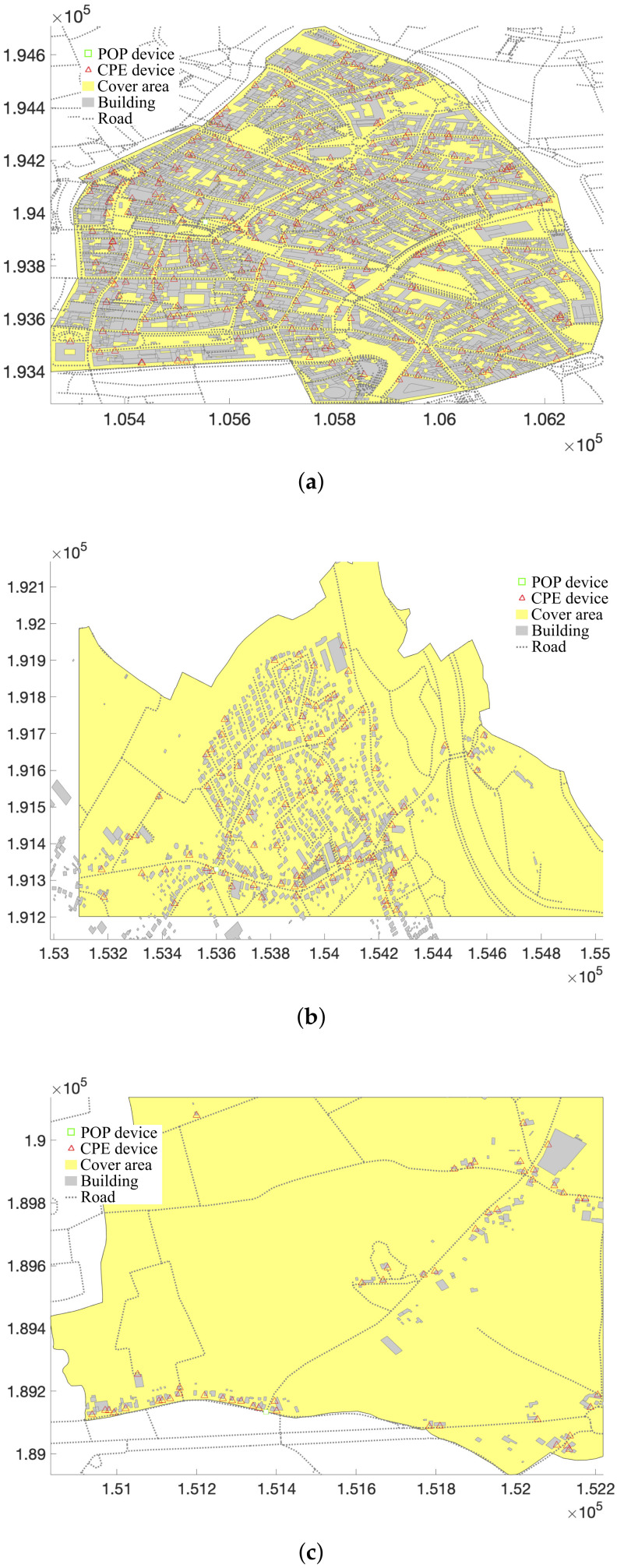
Simulation environments for FWA network characterization, with gray surfaces representing the buildings, dashed lines representing the roads, red triangles representing simulated customer premises equipment (cpe) locations, a green square representing the fiber point of presence (POP), and the considered surface area shown in yellow. (**a**) Urban city (Ghent). (**b**) Village (Leest). (**c**) Rural (Leest).

In this paper, we considered the scenarios for different FWA networks listed in Table 3. For each scenario, we ran 50 simulations with the GRAND tool to consider the randomness of the scenarios. Each simulation resulted in a database with cpe device locations and all of the LOS links between the devices. Three environments were compared, each having a surface area of 1 km${}^{2}$: downtown Ghent as an urban city, the village Leest, and a rural area in the neighborhood of Leest. The environments are shown in Figure 2. The number of cpe devices in the area, i.e., the internet subscribers to the network, ranged from 50 to 600. For the rural environment, only 10 and 50 cpe devices were considered, as there were fewer than 100 buildings. In the centralized pop scenario, one pop device was located centrally at a location where a cabinet currently exists. In the decentralized pop scenarios, two random cpe devices were replaced by a pop device. The weather condition changes from sunny to heavy rain, with a rain rate of 25 mm/h, and the influence of vegetation was analyzed assuming that 10% of the link distance is covered by vegetation.

For the user requirements, the two use cases presented in Table 4 were considered. In the first use case, it was assumed that all customers require a peak data rate of 300 Mbps. In the second use case, it was assumed that 30% of the customers subscribe to an economy plan (with a data rate of 30 Mbps), 30% of the customers subscribe to the standard plan (with a data rate of 100 Mbps), and 10% of the customers need a peak data rate of 500 Mbps.

Three different wireless communication technologies were considered: mmWave 5G at 28 GHz, IEEE Std. 802.11ad at 60 GHz, and then a future wireless communication system at 140 GHz. MmWave 5G operates from 26.5 GHz to 29.5 GHz and supports channel bandwidths up to 400 MHz. The minimum SNR ranges from 2.2 dB for binary phase shift keying (BPSK) modulation up to 25.2 dB for 256-QAM [60]. The maximum data rates that can be achieved were calculated via (6), with DR the data rate in Mbps, Q the modulation order that depends on the MCS, R the code rate, F the scaling factor (set to 1), N the maximum number of allocated resource blocks (set to 264, which corresponds to a subcarrier spacing of 120 kHz), T the symbol duration (calculated via $\frac{10^{-3}}{14\cdot 2^{n}}$ with n the numerology), and OH the overhead (set to 0.18 for frequency range 2 of the 5G specification) [61].

(6) $$\mathrm{DR}=Q\cdot R\cdot F\cdot \frac{12N}{T}\cdot \left(1-OH\right)\cdot 10^{-6}$$

In this equation, a SISO system with a single layer, i.e., a single data stream, is considered, in order to compare the results with the IEEE Std. 802.11ad technology. The selected code rates range from 0.5 (for BPSK) to 948/1024 (for 256-QAM) [61]. The numerology for a subcarrier spacing of 120 kHz is n = 3, and the corresponding data rates range from 145 Mbps for BPSK to 2.155 Gbps for 256-QAM for a channel bandwidth of 400 MHz.

At 60 GHz, IEEE Std. 802.11ad radios were considered, with channel bandwidths of 2.16 GHz and a single carrier physical layer. With code rates ranging from 0.5 for BPSK (MCS 1) to 0.75 for 16-QAM (MCS 12) and 0.81 for QPSK (MCS 9), the data rates range from 375 Mbps for BPSK to 4.62 Gbps for 16-QAM modulation, which requires an SNR of 12.6 dB [21]. An orthogonal frequency division multiplexing (OFDM) physical layer is also available with higher data rates, but was not considered for the remainder of this paper.

Currently, no wireless communication systems exist at 140 GHz. In order to compare future wireless communication systems at 140 GHz with existing technologies at 28 and 60 GHz, the channel capacity for the different frequencies was analyzed, which was calculated via (7), with C the channel capacity in bits/s, B the channel bandwidth in Hz, and SNR the signal-to-noise ratio.

(7) $$C=B\log_{2}\left(1+\mathrm{SNR}\right)$$

The channel capacity provides a theoretic upper bound of the spectral efficiency. For the comparison, the transmit power and antenna gains were kept constant, i.e., the same EIRP was considered for the three frequencies, and the difference in channel capacity was only caused by the different bandwidth and PL models for the three frequency bands. In reality, the EIRP of future wireless communication systems may differ because exposure regulations are subject to change, because the achievable transmit power of future systems is not yet determined, and because the used antenna systems may have larger antenna arrays with a high directivity.

## 4. Simulation Results and Discussion

### 4.1. Network Analysis

#### 4.1.1. Overview

A summary of the averaged network metrics for the different scenarios is presented in Table 5, considering a single pop device and without considering edge devices. The average cpe vertex degree $\delta_{v,\mathrm{avg}}$ increases when more cpe devices are present in the network. For the same number of cpe devices, the average cpe vertex degree decreases when more buildings are present, from 5.7 for a rural environment to 1.4 for a city center. The low POP eccentricity $\epsilon_{\mathrm{POP}}$ for an urban city with few cpe devices (50 or 100) can be explained by the large number of unconnected cpe devices, i.e., the cpe devices for which no route towards the POP exists before adding edge devices. The vertex characteristics of the pop device are critical to the success of the network deployment. The pop eccentricity is an important parameter for the latency, whereas the vertex degree influences the total network capacity, as all of the FWA network traffic is carried over one of the edges of the pop device. The median link distance d${}_{\mathrm{med}}$ gives an indication of the maximum throughput of the data that get transmitted over the wireless links. In the pilot deployment scenario in a village environment and in a rural environment, the median path length is larger and the capacity of the links will be lower. In an urban city, median link distances are limited to 80 m, irrespective of the number of cpe devices, as buildings obstruct links with a larger distance.

Analyzing the average percentage of connected cpe devices, i.e., the devices for which a path towards the pop exists irrespective of the link capacity, reveals that a single pop is not sufficient for a city environment. edge devices are critical for the FWA network deployment in urban cities. Adding edge devices is required in the field trial or early adopter phase for the village and rural environments, but with a sufficient number of network subscribers, the average percentage of connected cpe devices exceeds 86%. The average link distance increases as fewer buildings are present, ranging from 77 m for an urban city up to 247 m for a rural environment.

#### 4.1.2. Link Budget Calculations

The metrics from Table 5 are based on the input graph, i.e., the number and location of cpe devices and the number and placement of buildings. Therefore, they do not depend on the used frequency band, weather conditions, or obstructions due to vegetation. Based on the channel models presented in Section 2.2, and using the link budget calculations from Section 2.3, the maximum data rate on each link can be calculated based on the distances between FWA devices.

In an FWA network, there is bidirectional communication between identical devices. Therefore, the antenna gains of the transmit and receive antennas are the same. The total equivalent isotropically radiated power (EIRP), i.e., the sum of the transmit power and antenna gain, is subject to (local) regulations [56]. We performed link budget calculations for mmWave 5G and IEEE Std. 802.11ad. We considered no losses in the transmitting and receiving devices (L${}_{T}$ = L${}_{R}$ = 0 dB), and used an EIRP of 42 dBm for the two technologies. For mmWave 5G at 28 GHz, this EIRP can be obtained using an antenna gain of 19 dBi and a maximum transmit power P${}_{T}$ of 23 dBm. At 60 GHz, it is obtained using a transmit power of 10 dBm and an antenna gain of 32 dBi. The used EIRP corresponds to the maximum EIRP of the Terragraph IEEE Std. 802.11ad platform, consisting of 288 antenna elements [12].

The received power levels P${}_{R}$ were obtained via (5) and converted into SNR by subtracting the thermal noise floor, calculated via (8), with NF the noise floor in dBm, B the bandwidth in Hz, T the temperature in Kelvin, and k Boltzmann’s constant equal to 1.379 × 10${}^{-23}$ W Hz${}^{-1}$ K${}^{-1}$.

(8) $$\mathrm{NF}=10\log_{10}\left(\frac{\mathrm{kTB}}{1\mathrm{mW}}\right)$$

**Figure 3:**
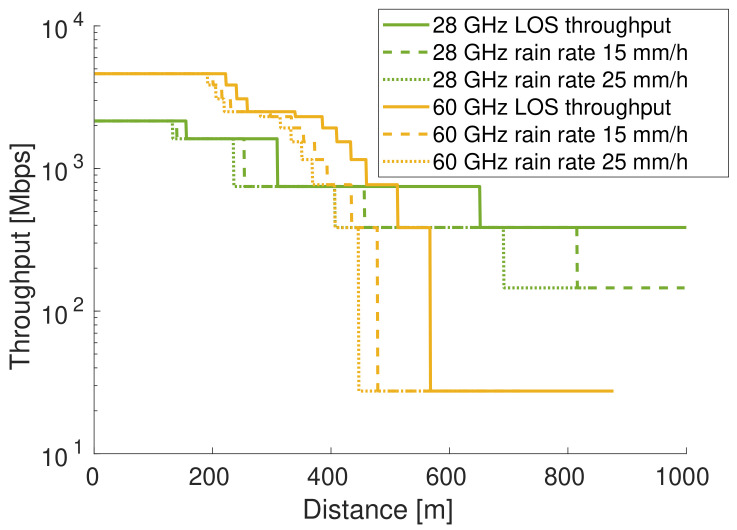
Maximum throughput as a function of distance for outdoor wireless networks at 28 GHz and 60 GHz.

Figure 3 shows the maximum link distance in meters as a function of the throughput for mmWave 5G and IEEE Std. 802.11ad technologies for a line-of-sight scenario and two scenarios with different rain intensities. As the EIRP for both technologies is equal, the differences in throughput are caused by the different PL models, and by different technology characteristics, such as the channel bandwidth. Even though the atmospheric loss and free space PL are higher at 60 GHz, larger data rates are obtained for link distances up to 500 m as the channel bandwidths at 60 GHz are larger. For larger distances, the increased PL at 60 GHz limits the SNR and mmWave 5G provides higher throughputs.

#### 4.1.3. Network Capacity

Table 6 presents the user requirements for the two considered use cases from Table 4. For use case 2, with different users requiring different data rates, the total user data rate requirement is lower than for use case 1, where all subscribers request 300 Mbps.

**Figure 4:**
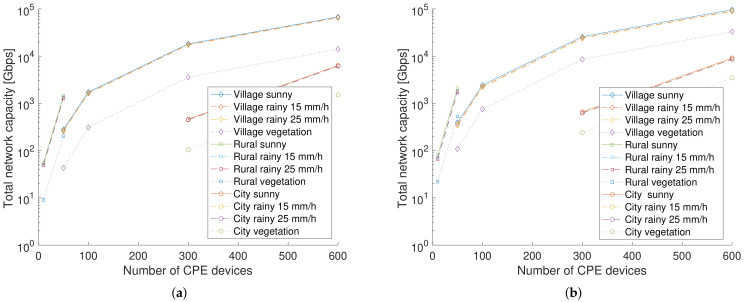
Averaged total network capacity as a function of the number of customer premises equipment (cpe) devices. (**a**) 60 GHz. (**b**) 140 GHz.

Figure 4 shows the total network capacity, i.e., the sum of the link capacities of all wireless links in the network calculated via (7), as a function of the number of cpe devices for different simulation configurations and averaged over the 50 simulations for frequencies of 60 GHz and 140 GHz. For the capacity calculations, all link budget parameters except PL and channel BW are identical. The total network capacity increases with an increasing number of cpe devices. For an urban city environment (represented by the circle symbols), the rain influence is limited, which is due to the smaller link distances. However, the total network capacity lowers by 66% for 60 GHz and 53% for 140 GHz when 10% of the links are covered by vegetation. Due to the higher average cpe vertex degree, the network capacity is higher for a village.

**Figure 5:**
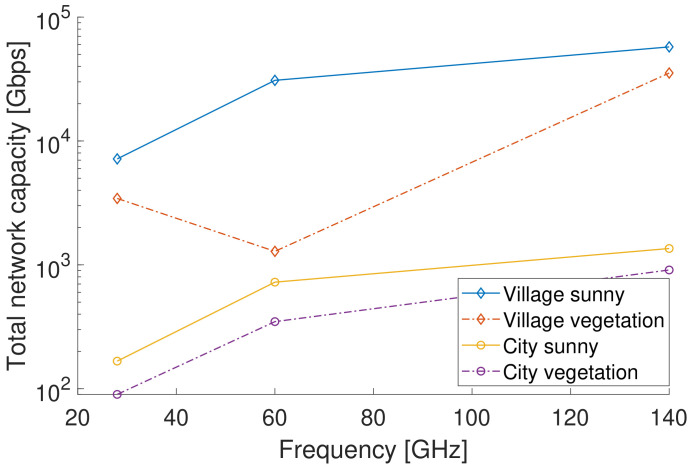
Averaged total network capacity as a function of carrier frequency for a village and city environment, with 300 network subscribers in an area of 1 km${}^{2}$.

Figure 5 shows the total network capacity as a function of frequency for the village and city environments, and considering scenarios with and without vegetation. Even though there is a higher PL when using higher frequencies, which results in a lower SNR, the larger bandwidths that are available result in a larger total network capacity. The impact of vegetation is most pronounced for 60 GHz, where a large attenuation has a considerable effect on PL and SNR.

Table 7 summarizes the influence of rain on the total available network capacity and average edge throughput that is available on the links for two environment scenarios, considering the IEEE Std. 802.11ad at 60 GHz, and comparing sunny weather with no additional attenuation to heavy rainfall with a specific attenuation of 10 dB/km. An early adopter scenario (with 100 cpe devices) was compared with a late majority scenario with 600 cpe devices. An investigation of the PL for the different wireless links shows that additional attenuation due to rain does not seem to be substantial, as the average link distances are small. The additional attenuation ranges from 0.1 dB for the smaller links to 3.7 dB for a link with a distance of 370 m. However, an additional attenuation of 3 dB has a considerable impact on the throughput that is available, as the receiver sensitivities for different MCS indices are close to one another [21]. For the early adopter scenario, the total network throughput, i.e., the sum of the throughputs of all wireless links, decreases by 12.5% and the average edge throughput decreases by 419 Mbps in the event of heavy rain. For the late majority scenario, the total network throughput decreases by 8% and the average edge throughput decreases by 277 Mbps. We conclude that the impact of rain on the late majority scenario is lower than for the early adopter scenario. This is caused by the smaller link distances for a denser scenario.

### 4.2. Network Planning

The goal of the network routing algorithm is to define a route from each cpe towards the pop, given a predefined QoS requirement, i.e., a certain throughput needs to be allocated on the edges from a cpe device to the pop device.

For the validation of the network planning algorithm, an IEEE Std. 802.11ad wireless system was considered, with a carrier frequency of 60 GHz and data rates of up to 4.6 Gbps for a single-carrier physical layer (PHY). Furthermore, we assumed that the required maximum download speed is 300 Mbps for each cpe, which corresponds to twice the typical download speed of a Belgian telecom operator [62].

#### 4.2.1. Network Planning Rural Environment

**Figure 6:**
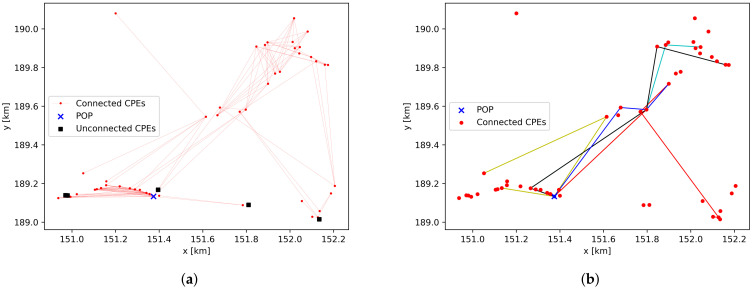
Graph representation and planning of a simulation in rural environment with 50 cpe devices, with the edge colors in (**b**) representing the paths from some cpe devices towards the pop device. (**a**) Graph representation. (**b**) Network planning.

The rural environment of Figure 2c was considered, with 50 cpe devices randomly distributed on the floor map. The graph representation of a single simulation result is shown in Figure 6a and has the following metrics. The average cpe vertex degree is 5.26, the pop eccentricity is 5.0, the median link distance is 199.7 m, and the average number of hops towards the pop is 2.3.

The input graph is not connected, as four cpe devices do not have a link to another device. These cpe devices are represented by a black square in Figure 6a, and an analysis of Figure 2c shows that they will be able to connect to neighboring devices if they are located at another facade of the same building. In ideal scenarios, i.e., no rain and no vegetation obstructing the LOS paths, all links have a throughput of 4.62 Gbps. This is in line with Figure 3, as the maximum link distance is less than 600 m. The sum of all user data rate requirements for use case 1, i.e., all users request 300 Mbps at the same time, is 15 Gbps, whereas the sum of all throughputs on the links to the pop is 73.92 Gbps. When it is raining with a rain rate of 25 mm/h, it is not possible to use the maximum data rate on all links (the maximum throughput of links exceeding 600 m decreases to 2.5 Gbps), and the sum of all throughputs towards the pop decreases to 67.374 Gbps. Therefore, the links towards the pop have enough capacity to transfer the data of all cpe devices. When 10% of the links are obstructed by vegetation, the available data rates decrease significantly, making communication impossible on most links. Only for link distances below 130 m is a data rate of at least 385 Mbps possible. For this scenario, the sum of the throughputs on the links towards the pop is 8.33 Gbps which is not sufficient for carrying all aggregated user data. Even when only 5% of all links are covered by vegetation, there is a capacity bottleneck on the links towards the pop, with an available throughput of 11.659 Gbps.

For defining the path from each cpe device towards the single pop device, the cpe devices are first sorted. All devices have a throughput requirement of 300 Mbps, and 80% of all devices have a single shortest path towards the pop. These devices are sorted based on the hop count on the shortest path, and network planning is performed first for the cpe devices with the largest hop count. Latency in multi-hop wireless networks is mainly caused by processing delays on the routing devices, i.e., the latency increases when more hops are present on the path. As the shortest path algorithm is used and the number of hops is minimized, the presented algorithm also optimizes the network for latency. For the scenario without any vegetation present, all cpe devices have a path towards the pop. Some examples are presented in Figure 6b, in which the red lines indicate the path with the largest hop count.

#### 4.2.2. Network Planning in Village Environment

**Figure 7:**
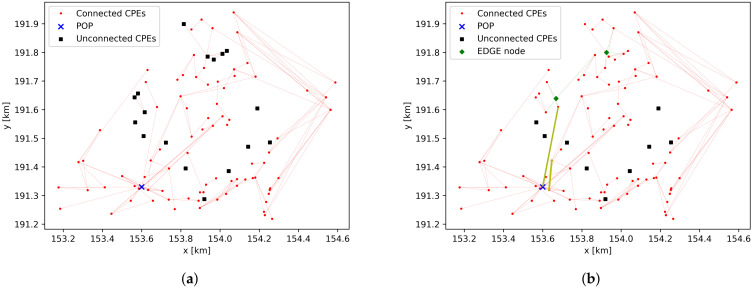
Graph representation of a simulation in village environment with 100 cpe devices. (**a**) Graph representation. (**b**) Two edge devices added.

In the pilot scenario (with 100 cpe devices) in the village environment, 16 cpe devices are not in the same cluster as the pop: 8 cpe devices do not have any connection and 2 clusters of 5 and 3 cpe devices, respectively, are interconnected but not connected to the main cluster. From the graph visualization in Figure 7a and the floor map of the environment in Figure 2b, it is clear that these devices can easily get connected by adding a limited number of edge devices to the FWA network. In Figure 7b, two edge devices are added at coordinates (153.668, 191.639) and (153.925, 191.800) that correspond to road intersections. Using this adjusted network, the network planning framework successfully performs network planning for the first 49 cpe devices before another warning is shown. From Figure 7, it is clear that the number of links from the pop going north is limited, and this causes capacity constraints on the links that are plotted in yellow in Figure 7b. To resolve the capacity constraint, edge devices can be added near coordinates (153.960, 191.560) and (153.660, 191.500). These edge devices create additional paths from the cpe devices in the north region towards the pop device. Another possibility is to investigate the placement of an additional pop device in the north region of Leest.

#### 4.2.3. Discussion

The cpe devices have a small number of shortest paths to the pop device, and the number of hops on the shortest path is limited. The large bandwidths in the mmWave frequency band realize wireless networks where enough capacity is available on the wireless links to serve all users with a data rate that exceeds the data rate of current wired access networks. For a village and rural environment, up to five edge devices need to be added to make the graph connected, i.e., to enable a connection from each cpe device to the pop device. For an urban city environment, more pop devices are required to get the cpe devices connected to the FWA network. Taking into account a vegetation map (with an indication of where trees are located) will allow for a more realistic link capacity calculation.

Different routing algorithms were implemented and tested, but resulted in a sub-optimal solution. As an example, when multiple connected cpe devices are first combined and network routing is performed for the single *virtual* cpe device, the required data rate of the *virtual* cpe device increases and bottlenecks appear that are not present when performing network routing for all individual cpe devices. The optimal solution for the network routing of internet protocol data is the Busacker–Gowen algorithm, which is an algorithm used to solve minimum cost maximum flow problems [63]. In the Busacker–Gowen algorithm, data are routed from a user towards a server via multiple paths. The benefit of the proposed network planning algorithm compared to the Busacker–Gowen algorithm is that data are not split between multiple paths, i.e., all data from a certain cpe device follow the same path, even for multiple subscribers with different data rate requirements. This makes the presented implementation more suited for the planning of real FWA networks. In the current implementation, no beam steering loss and beam switching are considered, i.e., the angles of arrival and departure of the different links are not taken into account.

Compared to the FWA network planning solution presented in [17], where the cpe devices have no routing capabilities, very limited edge devices are required to enable network routing from all cpe devices towards the pop device. The advantage of the current approach is that fewer edge devices are required, which makes the network easier to deploy, as the placement of radio equipment on public buildings and street furniture requires significant administrative work. The drawback is that cpe devices with routing capabilities require a more complex RF and networking architecture. This makes the cpe devices more expensive compared to cpe devices that only need a single wireless connection towards an edge device. In [17], 116 edge devices are required to cover 100 cpe devices (without routing capabilities) in the village of Leest. As such, the total installation cost of the cpe and edge devices for a telecom operator will be lower using our proposed network architecture.

## 5. Conclusions

In this paper, we used graph theory to analyze the architecture of FWA networks adopting realistic channel models for performing reliable link budget calculations. Furthermore, we presented a routing algorithm used to define how cpe devices can route their internet data traffic towards the pop device that connects to the wired infrastructure.

Some graph metrics, including the average vertex degree and the average path length, influence the capacity of the network, whereas other metrics, e.g., the average hop count, influence wireless system characteristics such as latency. Due to the high vegetation loss at mmWave frequencies, capacity bottlenecks occur when even a small part of the link, i.e., 5% or 10%, is obstructed by vegetation. Due to the short link distances, the rain attenuation does not have a considerable effect. The larger bandwidths that are available at higher frequencies, 400 MHz at 28 GHz versus 2.16 GHz at 60 GHz, enable larger data rates. The influence of the high atmospheric loss at 60 GHz is limited due to the small link distances.

For the validation of the proposed network planning algorithm, an example is provided to perform FWA network planning using IEEE Std. 802.11ad technology operational in the 60 GHz band. The network planning determines the route from each cpe device towards the pop device, considering the channel capacities of the wireless links. When certain cpe are not connected to the network or the available capacity is not sufficient, edge devices need to be added to the network. The edge devices act as a router and do not connect customers directly. For urban city environments, the edge devices are critical to get cpe devices connected to the network. In this work, the placement of edge devices was considered to be a manual task that is performed by the network operator. Based on the location where the capacity bottleneck occurs, and based on the local regulations, the operator adds edge devices to increase the capacity. It was shown that a limited number of edge devices is sufficient for rural and village environments.

Theoretic channel capacity calculations at three frequencies were compared, including 140 GHz, where no wireless technologies currently exist at that frequency. An assessment of the assumptions made in this work, e.g., the actual EIRP of a system operating at 140 GHz, can be performed when new wireless systems become available.

In the current work, a constant antenna gain was considered for all beam-forming angles. Future work includes analyzing beam steering loss and realistic antenna beam patterns for cpe devices that maintain multiple links. In addition, non-line-of-sight links can be considered, e.g., by searching for paths with a single building reflection and using typical reflection loss values from the literature. The implementation of in-band telemetry monitoring allows for changing network configurations to maintain QoS [64] when the environment changes, e.g., when new buildings are built, when vegetation is added, or when devices in the network are broken. On higher layers, scheduling and medium access control mechanisms need to be investigated.

## Figures and Tables

**Table 1:** One-slope path loss (PL) model parameters for different frequencies, with reference distance d${}_{0}$ = 1 m.

Frequency	PL${}_{0}$	n	σ	Reference
28 GHz	61.4 dB	2.1	3.6 dB	[38]
60 GHz	71.0 dB	1.8	2.9 dB	[12]
140 GHz	75.9 dB	1.9	1.4 dB	[13]

**Table 2:** Specific attenuation in dB/km for two rain rate intensities at frequencies of 28 GHz, 60 GHz, and 140 GHz.

Rain Rate	28 GHz	60 GHz	140 GHz
15 mm/h	2.4 dB/km	6.8 dB/km	9.0 dB/km
25 mm/h	3.9 dB/km	9.5 dB/km	12.6 dB/km

**Table 3:** Considered scenarios for FWA network analysis.

Parameter	Scenarios
Surface area	1 km${}^{2}$
Number of cpe devices	50 (field trial network)
	100 (early adopter)
	300 (early majority)
	600 (late majority)
Number of pop devices	1 (central pop)
	3 (decentralized pop)
Environment	urban city (Ghent)
	village (Leest)
	rural (Leest)
Weather condition	sunny (no rain)
	rain (15 mm/h)
	heavy rain (25 mm/h)
Vegetation obstruction	none
	10% of the link is covered by vegetation

**Table 4:** Considered throughput requirements of the network subscribers.

Use Case	30 Mbps	100 Mbps	300 Mbps	500 Mbps
1	0%	0%	100%	0%
2	30%	30%	30%	10%

**Table 5:** FWA network characterization for different environments and network densities, with $\delta_{v,\;\mathrm{avg}}$ the average cpe vertex degree, $\epsilon_{\mathrm{POP}}$ the POP eccentricity, d${}_{\mathrm{med}}$ the median link distance in meter, l${}_{\mathrm{avg}}$ the average path length towards the pop in hop count, and c${}_{\mathrm{avg}}$ the average percentage of connected cpe devices without adding any edge devices.

Environment	# cpe	$\delta_{v,\mathrm{avg}}$	$\epsilon_{\mathrm{POP}}$	d${}_{\mathrm{med}}$	l${}_{\mathrm{avg}}$	c${}_{\mathrm{avg}}$
Urban city	50	1.43	1.40	77.80 m	1.18	3.6%
Urban city	100	2.10	2.12	80.45 m	1.50	2.9%
Urban city	300	3.48	6.36	78.46 m	3.43	5.7%
Urban city	600	4.63	14.72	77.37 m	7.19	23.8%
Village	50	2.39	4.14	136.3 m	2.15	31.5%
Village	100	3.35	8.24	116.7 m	3.37	65.3%
Village	300	7.30	6.94	106.1 m	3.22	97.5%
Village	600	12.86	6.02	99.2 m	3.36	98.8%
Rural	10	2.00	1.50	247.3 m	1.21	45.0%
Rural	50	5.68	4.50	214.6 m	2.10	86.4%

**Table 6:** Total user data rate requirement for two use cases as a function of number of cpe devices.

Use Case	10 cpe	50 cpe	100 cpe	300 cpe	600 cpe
1	3 Gbps	15 Gbps	30 Gbps	90 Gbps	180 Gbps
2	1.8 Gbps	9.0 Gbps	17.9 Gbps	53.7 Gbps	107.4 Gbps

**Table 7:** Influence of rain attenuation on total and average (AVG) network throughput of an IEEE Std 802.11ad system at 60 GHz.

Scenario	Weather	Total Throughput	AVG Edge Throughput
100 cpes	Sun	411.94 Gbps	3.35 Gbps
100 cpes	Rain rate 25 mm/h	360.50 Gbps	2.93 Gbps
600 cpes	Sun	13,386.84 Gbps	3.50 Gbps
600 cpes	Rain rate 25 mm/h	12,326.43 Gbps	3.23 Gbps

## Data Availability

The channel measurement data discussed in this study have been made available via the NextG database (accessible via http://nextg.nist.gov/ (accessed on 28 November 2022)) and via Datadryad at https://doi.org/10.5061/dryad.d2547d85n (accessed on 28 November 2022).

## Abbreviations

The following abbreviations are used in this manuscript:

AVG	Average
BPSK	Binary phase shift keying
CMOS	Complementary metal–oxide–semiconductor
CPE	Customer premises equipment
DR	Data rate
DSL	Digital subscriber line
EIRP	Equivalent isotropically radiated power
FinFET	Fin field-effect transistor
FWA	Fixed wireless access
GRAND	Green radio access network design
IEEE	Institute of electrical and electronics engineers
IF	Intermediate frequency
ISM	Industrial, scientific, and medical
ITU	International telecommunication union
LAN	Local area network
LOS	Line-of-sight
LTE	Long-term evolution
MAC	Medium access control
MCS	Modulation and coding schemes
MDPI	Multidisciplinary digital publishing institute
MIMO	Multiple-input multiple-output
mmWave	Millimeter-wave
OFDM	Orthogonal frequency division multiplexing
PHY	Physical layer
PL	Path loss
POP	Point of presence
SISO	Single-input single-output
SNR	Signal-to-noise ratio
QAM	Quadrature amplitude modulation
QoS	Quality of service
VED	Vegetation-dependent exponential decay
QoS	Quality of service
VED	Vegetation-dependent exponential decay

## Appendix A. Network Modeling and Planning Framework

The framework for FWA network characterization and network planning was implemented in Python and has been made available on GitHub: https://github.com/brdb/fwa-network-modeling-and-planning (accessed on 12 February 2023). In this appendix, a brief overview of the code structure is provided. For a detailed presentation, we refer to the README.md file in the repository. The repository has the following file structure.

The simulation data from the GRAND tool, discussed in Section 3.3, for 50 simulations can be found in the data directory for the different scenarios discussed in this paper (Table 3). In the file graph_creation from the core directory, the input data are parsed and a graph is constructed using the Python *iGraph* package. The vertices represent cpe devices, and the edges represent line-of-sight links between the devices. In the file graph_extension, the file with edge device locations and links is parsed and the edge devices are added to the graph. The characterization of an input graph, presented in Section 4.1.1, is performed via the file graph_analysis. The file network_planning implements the network planning algorithm from Section 3.2. Helper functions for the visualization of graphs and for the link budget calculations used to transform the link distance into available throughputs and link capacities are present in the utils directory. In the examples directory, scripts are presented for the analysis and network planning of different scenarios, as an example of how to use the presented framework. The script graph_analysis_overview generates the data from Table 5. The data from Figure 4 are generated via network_capacity_overview, and the data from Table 7 are generated via rain_influence_analysis. The scripts used to illustrate the network planning algorithm are UC1_network_planning_ 100CPE_UrbanVillage and UC1_network_planning_50CPE_ Rural. The scripts directory contains MATLAB scripts that are used for the visualization of the results and to create the figures presented in this paper. The data from Table 4 are generated from the MATLAB script visualize_required_capacity. The Python scripts print relevant information to the console terminal, whereas debugging information is written to a .log file.

The framework also contains an implementation of a graph simplification algorithm by combining connected vertices into cliques and perform network planning for cliques rather than individual cpe devices. However, this strategy introduces additional bottlenecks, i.e., there might be a bottleneck to route aggregated data from three cpe devices (e.g., 3 × 300 Mbps) to the pop device, whereas three routes with 300 Mbps exist. As this strategy is sub-optimal, it is not presented in this paper.
